# Projected impact of fast-tracking of anti-retroviral treatment coverage on vertical transmission of HIV in India

**DOI:** 10.1371/journal.pgph.0003702

**Published:** 2024-09-18

**Authors:** Pradeep Kumar, Chinmoyee Das, Subrata Biswas, Nidhi Priyam, Lalit Singh Kharayat, Damodar Sahu, Sanjay K. Rai, Sheela V. Godbole, Elangovan Arumugam, P. V. M. Lakshmi, Shanta Dutta, H. Sanayaima Devi, Arvind Pandey, Dandu Chandra Sekhar Reddy, Sanjay Mehendale, Shobini Rajan

**Affiliations:** 1 National AIDS Control Organization, Ministry of Health and Family Welfare, New Delhi, India; 2 Indian Council of Medical Research, National Institute of Medical Statistics, New Delhi, India; 3 All India Institute of Medical Sciences, New Delhi, India; 4 Indian Council of Medical Research, National AIDS Research Institute, Pune, India; 5 Indian Council of Medical Research, National Institute of Epidemiology, Chennai, India; 6 Postgraduate Institute of Medical Education and Research, Chandigarh, India; 7 Indian Council of Medical Research, National Institute of Cholera and Enteric Diseases, Kolkata, India; 8 Regional Institute of Medical Sciences, Imphal, India; 9 Indian Council of Medical Research, New Delhi, India; 10 Institute of Medical Sciences, Banaras Hindu University, Varanasi, India; 11 PD Hinduja Hospital and Medical Research Center, Mumbai, India; The AgaKhan University, PAKISTAN

## Abstract

One of the five high-level goals under Phase V of the National AIDS and STD Control Programme (NACP) of the Government of India is the elimination of vertical transmission of HIV. In this paper, we estimate the potential impact of maintaining and enhancing the anti-retroviral treatment under the NACP in terms of averting new infections and vertical transmission rates vis-à-vis no intervention scenario. We used India’s HIV Estimates 2022 models to create treatment coverage scenarios of no interventions, status quo, business as usual, on-track and fast-track scenarios from 2023 to 2030. Our analysis indicates that fast-tracking scale-up of treatment services would avert almost 41000 child infections from 2023 to 2030 leading to a vertical transmission rate of around 7.70% in 2030 vis-a-vis no interventions scenario. Higher and sustained ART coverage would not only take the country closer to the elimination goals but would also prevent thousands of vertical transmissions, thus bringing a lot of benefits to HIV-positive pregnant women and their families. Supported by efforts for the prevention of new infections in the general population, India is on track for the attainment of elimination of vertical transmission of HIV by 2030.

## Introduction

Countries around the world are committed to attaining the elimination of vertical transmission of HIV (EVTH), *inter alia*, as a core component of reaching the 95-95-95 targets by 2025 and ending AIDS as a public health threat by 2030 [1,2]. The elimination target aims to have at least 95% anti-retroviral therapy (ART) coverage for pregnant women living with HIV. Attainment of this treatment target would result in a vertical HIV transmission (VHT) rate of <2% in non-breastfeeding populations OR <5% in breastfeeding populations, a key impact benchmark [3].

In many circumstances, these targets are considered aspirational with only 16 countries validated for EVTH till 2023 [4,5]. However, world leaders and UN organisations have advocated for higher benchmarks to respond to the elimination potential of vertical transmission. As a result, more and more countries have started to include EVTH-related indicators under high-level targets and goals; India being one among them [6–10].

Presently, phase V of the National AIDS and STD Control Programme (NACP Phase-V) is under implementation in India. The phase has been designed through a consultative process involving various stakeholders and is funded through the domestic budget of the Government of India. It has five high-level goals and 23 output/outcome targets to anchor the national response till 2025–26. Elimination of vertical transmission of HIV and Syphilis is explicitly stated as Goal 3 of NACP Phase-V.

India’s efforts for prevention and elimination of vertical transmission of HIV have been anchored by a comprehensive four-pronged strategy. This strategy emphasizes the primary prevention of HIV (prong 1), the prevention of unintended pregnancies (prong 2), the prevention of vertical transmission from women living with HIV to their babies (prong 3), and the provision of care, support, and treatment for women living with HIV and their children (prong 4) [11]. Among all, prong 3 is the most critical to impact the vertical transmission rates [12]. While noteworthy progress has been made under the NACP on the road to elimination, there is still a long way to go with ART coverage among HIV-positive women at 77% and VHT rate at 19.9% [13].

In this paper, we aim to quantify the impact of the incremental changes to the current treatment coverage of HIV-positive pregnant women on the vertical transmission rate using the UNAIDS-recommended ‘Spectrum’ modelling tool. We also aim to understand the epidemiologic impact of these changes on vertical infections averted between 2023 and 2030 for incremental changes. The paper is intended to provide evidence to policymakers, funders, and development partners in funding scaled-up interventions while firming up the roadmap towards the attainment of EVTH.

## Methods

### HIV burden estimations in India

HIV burden estimation in India is done using the DemProj (Demography) and AIDS Impact Model (AIM) modules of the Spectrum Policy Modeling Software [14]. Under the direction of the UNAIDS Reference Group on Estimates, Modeling, and Projections, the AIM module is updated regularly. The reference group’s website (www.epidem.org) and the software developer’s website (https://avenirhealth.org/software-spectrum.php) provide the details of the updates.

In India, the models are prepared for each State and Union Territory (UT) separately. Overall, 34 models are developed. DemProj module is used to project the population for each State/UT by age and sex, based on assumptions about fertility, mortality, and migration. After projecting the demographics, the AIM module is used to project the consequences of the HIV epidemic, including the number of people living with HIV, new infections, and AIDS deaths by age and sex, as well as the need of the services for elimination of vertical transmission. We used the State/UT-specific model prepared for the 2022 round through Spectrum 6.24 of HIV Estimations in India under NACP as a starting point for developing the scenario for treatment coverage between 2023 and 2030 and then used the ‘Aggregate Uncertainty tool’ for State/UTs models in each scenario to model the national-level impact of each scenario on the vertical HIV transmission rate.

### Vertical transmission

The details for the method, including inputs and calculations, of the vertical transmission rate under the AIM module of the Spectrum have been described elsewhere [15–18]. In brief, demographic, programmatic, and HIV prevalence data among people aged 15 to 49 is first entered into the Model. The model then uses the input data, along with assumptions about CD4 progression and survival both on and off anti-retroviral treatment (ART), to convert prevalence trends into incidence trends. Based on data from community-based surveys or the type of epidemic, the incidence estimates are then broken down by age and sex to estimate the size of people living with HIV by sex and by age group.

Fertility rates for women living with HIV (WLHIV) in the 15–49 age group are modified to account for the effects of HIV infection. The adjusted overall fertility rates and the age distribution of fertility in each age group are multiplied by the number of WLHIV in that age group to estimate WLHIV in need of EVTH-related services.

**Fig 1:**
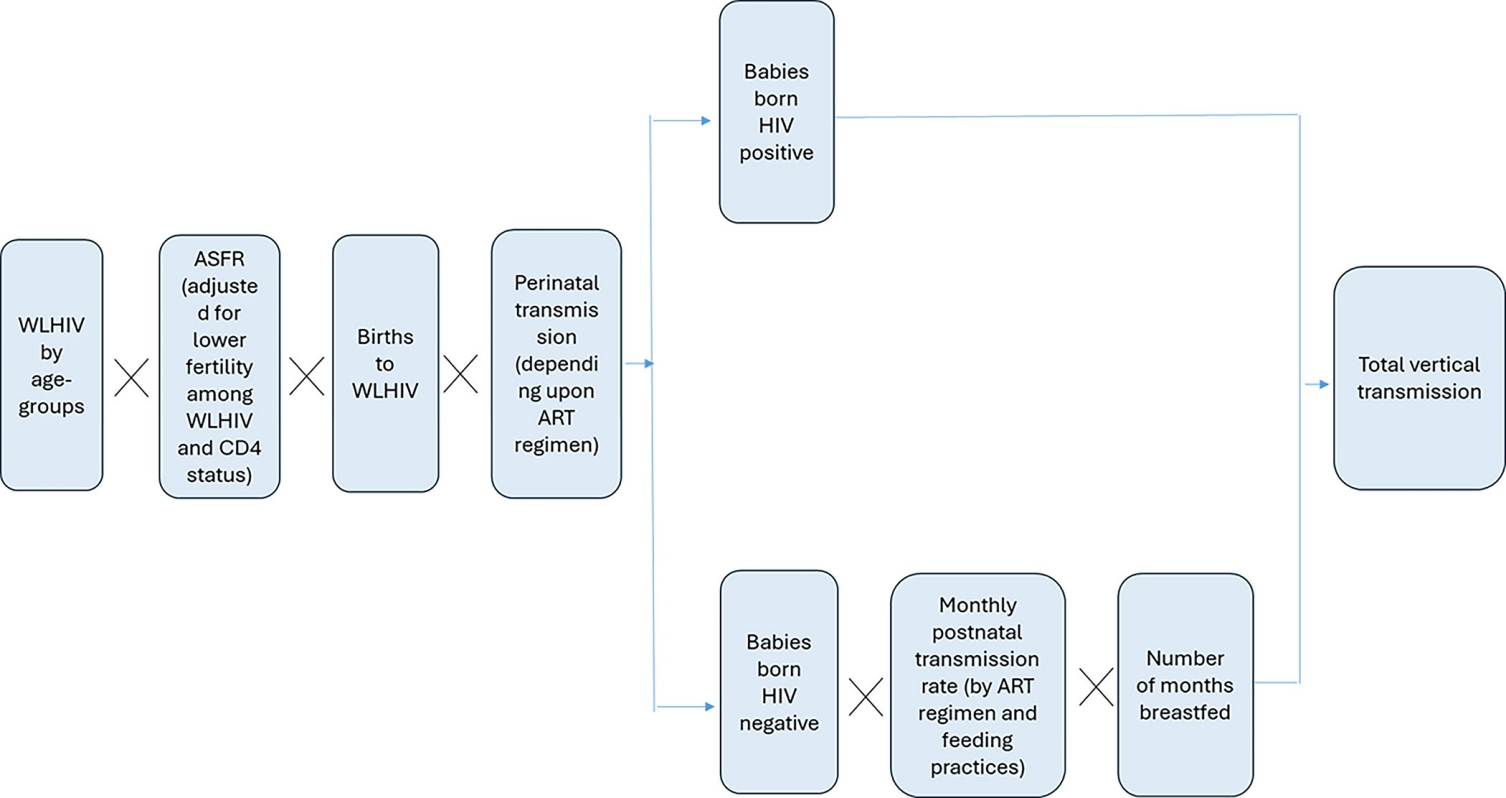
Summary of calculation for estimation of vertical transmission of HIV in Spectrum.

The model then estimates the vertical transmission of HIV depending upon the transmission probabilities which vary by coverage of the prophylaxis/treatment group separately for the pregnancy and delivery period (perinatal) and breastfeeding (postnatal) period. For each month the infant is breastfed, a monthly transmission probability is applied to estimate the cases of postnatal transmission except for the incident infections. The perinatal and postnatal transmission probabilities for different ART regimens and different CD4 levels of the mothers have been presented in Table 1. Fig 1 summarizes the calculation of vertical transmission [15].

**Table 1:** Perinatal and postnatal transmission probabilities by antiretroviral drug (ARV) regimens.

Regimen	Perinatal	Breastfeeding (per month)	
		< 350	> = 350
No prophylaxis			
Existing infections			
CD4 < 200	37	0.89	
CD4 200–350	27	0.81	
CD4 > 350	15		0.51
Incident infections	18.1	26.9 (one-time event)	26.9 (one-time event)
Single dose nevirapine	7.5	0.99	0.4
WHO 2006 dual ARV regimen	2.2	0.18	0.18
Option A	4.1		0.2
Option B	1.9		0.13
ART			
Started before pregnancy	0.26	0.02	
Started during pregnancy > 4 weeks	1.4	0.11	
Started during pregnancy < 4 weeks	8.2	0.2	

### Scenarios

For the present study, we developed five scenarios of treatment coverage and retention rate between 2023 and 2036: No intervention scenario (SC0), Status quo scenario (SC1), business as usual scenario (SC2), on-track scenario (SC3), and fast-track scenario (SC4) (Table 2). In each intervention scenario, we kept the rates stable once the level of 95% was achieved. State/UT-wise coverage in each scenario is available in supplementary tables. Comparisons were made against the status quo scenario vis-à-vis different intervention scenarios to demonstrate the impact.

**Table 2:** Treatment and retention coverage by scenario.

Scenario	Description
No intervention (SC0)	No ART is provided to prevent vertical transmission at any point.
Status quo (SC1)	ART treatment coverage and retention rate kept at their 2022 values throughout the projection period of 2023 to 2036.
Business-as-usual (SC2)	ART treatment and retention coverage is projected for the future years with 5% increase every year from the value of 2022. Rates were kept stable for future years once the level of 95% was achieved.
On-track (SC3)	ART treatment and retention coverage linearly modified to attain 95% by 2027. Rates were kept stable for future years once the level of 95% was achieved.
Fast-track (SC4)	ART treatment and retention coverage linearly modified to attain 95% by 2025. Rates were kept stable for future years once the level of 95% was achieved.

In no intervention scenario (SC0), no ART was provided for the elimination of vertical transmission across the projection period. This scenario predicts vertical transmission assuming that ART has never been made available to prevent vertical transmission. This scenario forms the base scenario to quantify the impact of each intervention scenario.

The status quo scenario (SC1) assumes the ART treatment coverage and retention rate at their 2022 values throughout the projection period of 2023 to 2036. State/UT-wise coverage in each scenario is available in S1 Table.

In the business-as-usual scenario (SC2), we increased treatment coverage by 5% per year, consistent with national increase in treatment coverage between 2021 and 2022, from 2022 onwards with stabilization at 95% once achieved. State/UT-wise coverage in scenario 2 is available in S2 Table.

For the on-track scenario (SC3) and fast-track scenario (SC4), coverage and retention rates were linearly modified to attain 95% by 2027 and 2025 respectively with the rates stable afterwards. State/UT-wise coverage in scenarios 3 and 4 is available in S3 and S4 Tables.

We aggregated State/UT models of each scenario using the ‘Aggregate Uncertainty tool’ of Spectrum to develop the national-level impact projections for the given scenario. For the national model in each scenario, we generated the number of new child infections due to vertical transmission and vertical transmission rate (including that of the breastfeeding period) for each of the projection periods. To assess the impact of the different scenarios, the fraction of vertical HIV infections prevented over 2023 to 2030 was estimated by comparing the cumulative number of vertical infections between each specific scenario vis-à-vis no intervention scenario (SC0). The vertical transmission rate refers to the percentage of children born to HIV+ mothers who will eventually be infected through vertical routes and includes perinatal transmission and up to 36 months of breastfeeding.

### Ethics statement

Surveillance of HIV and related co-infections is an essential component of the range of strategic information-related activities conducted by the Government of India’s National AIDS Control Organization (NACO) under the NACP [19]. Using the integrated and enhanced surveillance & epidemiology (IESE) framework and engaging ten government public health institutes, this activity is carried out following the provisions under the HIV & AIDS (Prevention and Control) Act, 2017 of the Government of India. The participating institutes present the technical framework for activities under IESE to their separate ethical committees for approval. Participants’ informed consent is accordingly obtained for bio-behavioural data collection and subsequent analysis to inform the national programme. After every round of surveillance, NACO estimates the HIV burden using aggregated, de-identified data. The results of the HIV burden exercise are published periodically [20–22]. As this study used aggregated de-identified outputs generated through the HIV Estimations 2022 model, ethical approval was not required.

## Results

**Fig 2:**
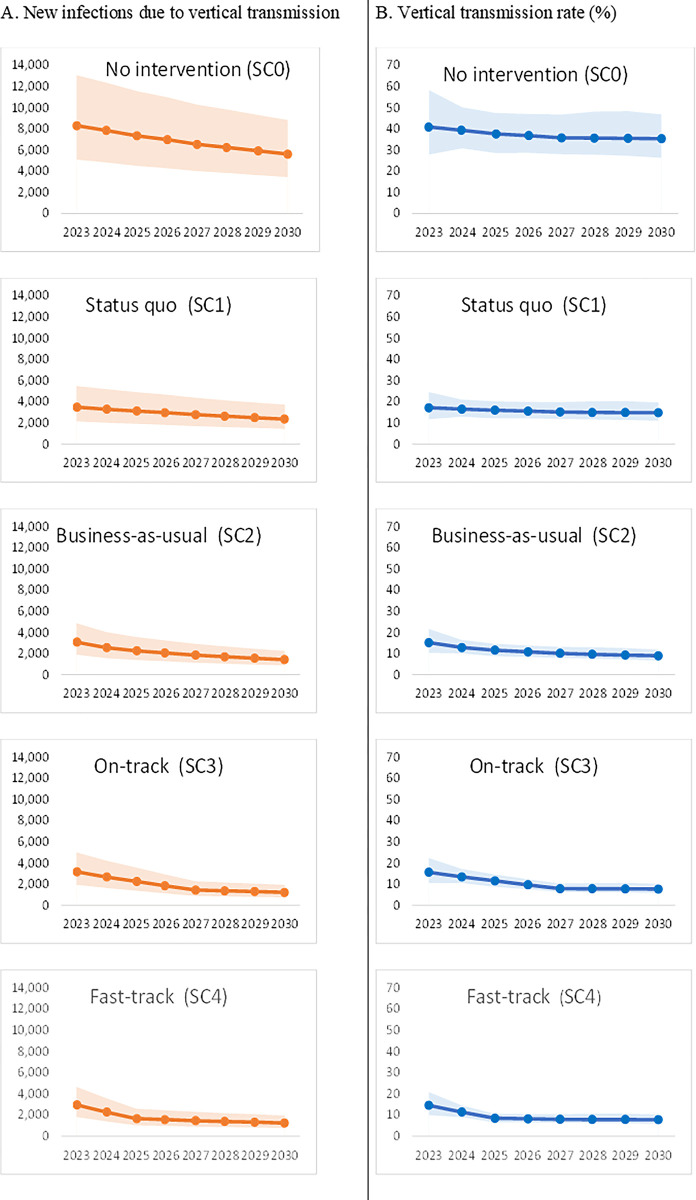
New infections and vertical transmission rate by scenarios.

**Fig 3:**
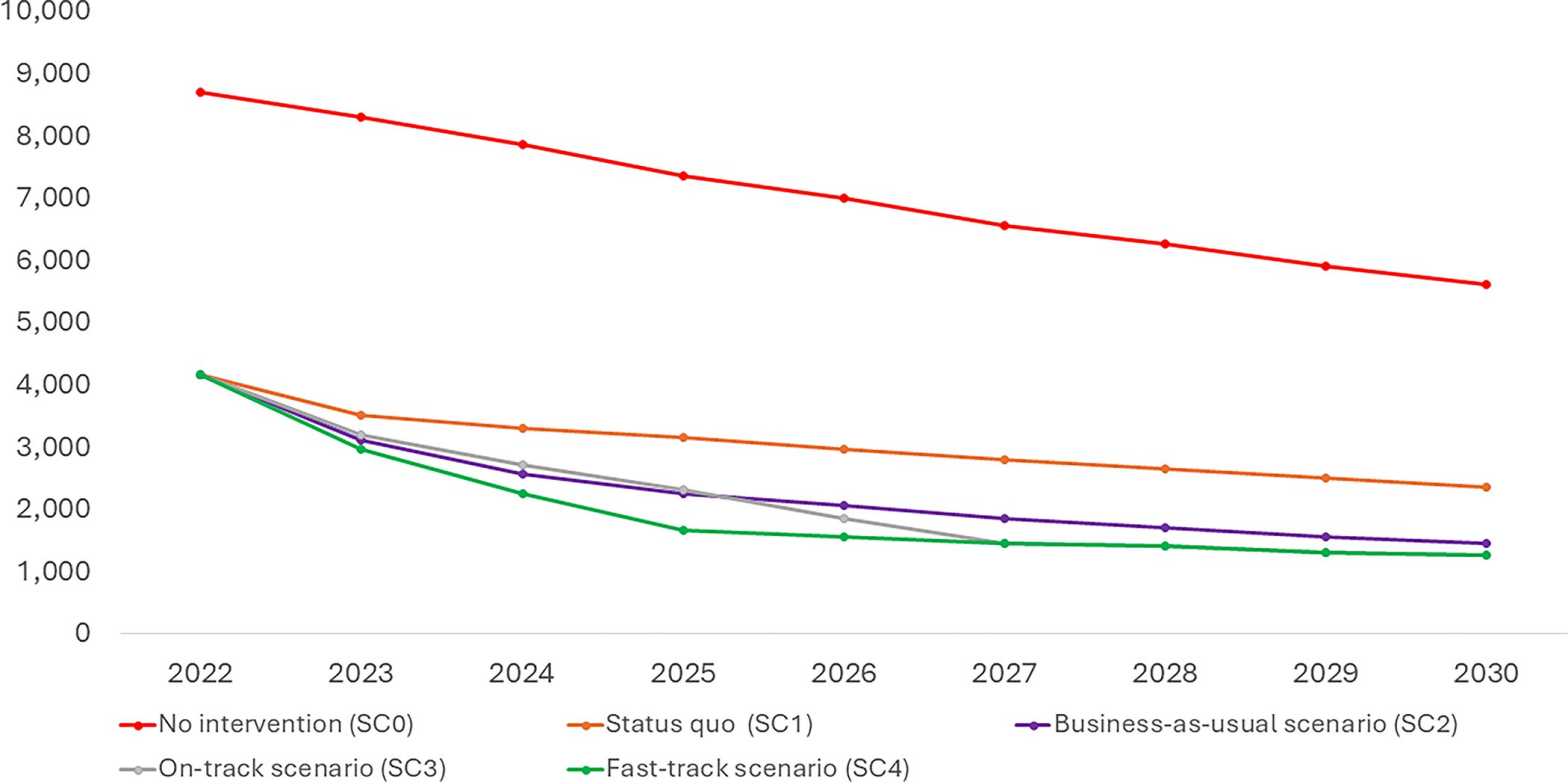
New HIV infections through vertical transmission in different scenarios.

**Fig 4:**
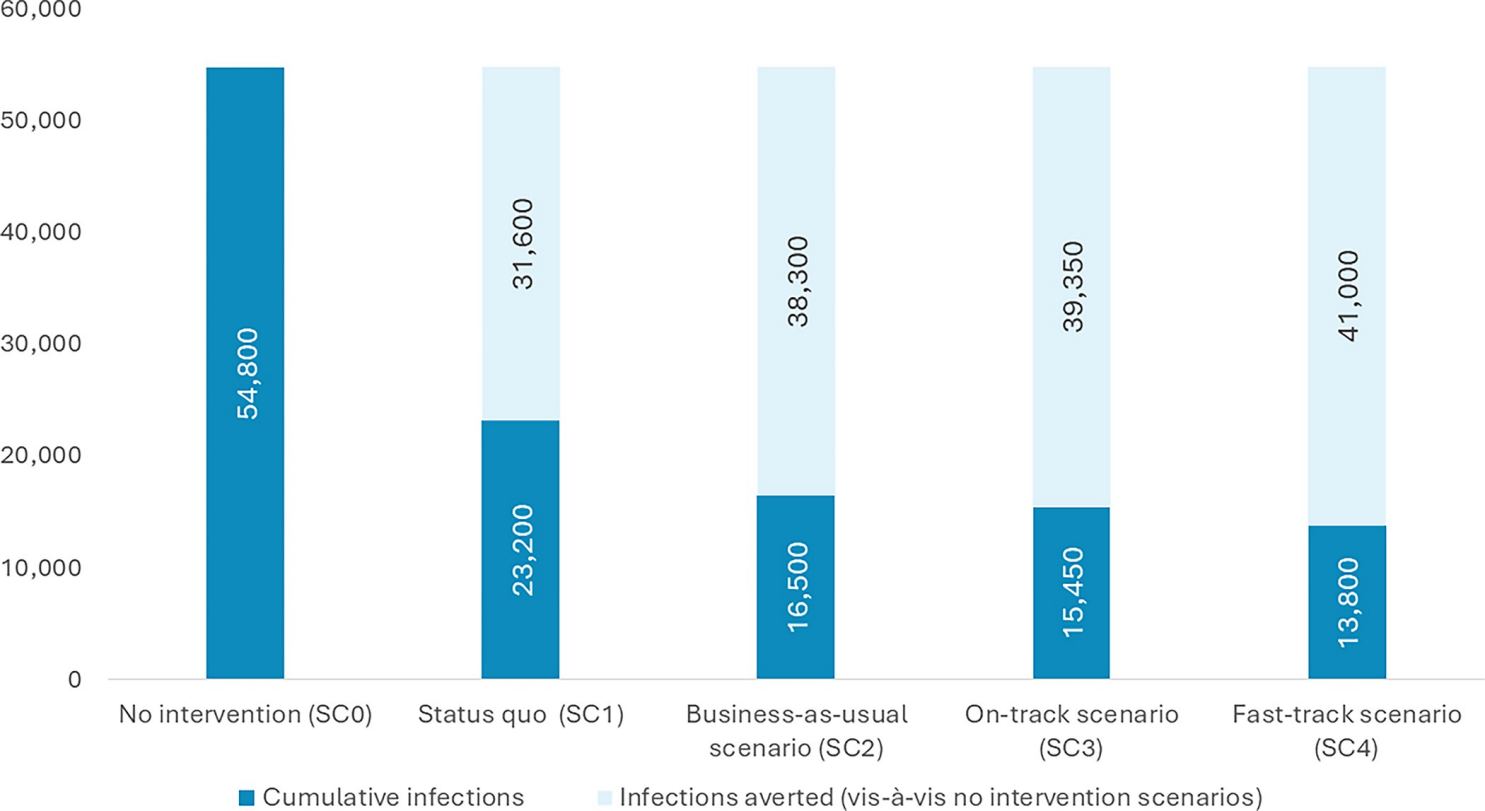
Cumulative number of new HIV infections through vertical transmission and infections averted between 2023 and 2030 in different scenarios.

In the absence of any anti-retroviral therapy (ART) for HIV-positive pregnant women, new infections through the vertical route would decline from 8,300 (5,100–13,050) in 2023 to 5,600 (3,450–8,800) in 2030 while the vertical transmission rate would decline from 40.80% (27.90%-58.30%) to 35.30% (26.30%-46.80%). Approximately 54,800 (33,600–86,150) new infections through the vertical route will happen in 8 years between 2023 and 2030 if no ART is being provided to HIV-positive pregnant women (No intervention scenario, SC0) (Table 3 and Figs 2–4).

**Table 3:** New infections and vertical transmission rate by scenarios.

Indicator	Year								
	2022	2023	2024	2025	2026	2027	2028	2029	2030
**No intervention (SC0) scenario**									
New child infections due to vertical transmission	8700 (5350–13700)	8300 (5100–13050)	7850 (4800–12350)	7350 (4500–11550)	7000 (4300–11000)	6550 (4000–10300)	6250 (3800–9800)	5900 (3650–9300)	5600 (3450–8800)
Vertical transmission rate (%)	41.9 (25.2–64.6)	40.8 (27.9–58.3)	39.3 (30.8–50.2)	37.6 (28.5–47.4)	36.8 (28.6–47)	35.7 (27.9–46.8)	35.6 (27.8–48.1)	35.5 (27.3–48.3)	35.3 (26.3–46.8)
**Status quo (SC1) scenario**									
New child infections due to vertical transmission	4150 (2550–6500)	3500 (2150–5500)	3300 (2000–5200)	3150 (1900–4950)	2950 (1800–4700)	2800 (1700–4400)	2650 (1600–4150)	2500 (1550–3950)	2350 (1450–3750)
Vertical transmission rate (%)	19.9 (12–30.7)	17.2 (11.8–24.6)	16.5 (12.9–21.1)	16 (12.1–20.2)	15.6 (12.2–20)	15.2 (11.9–19.9)	15 (11.7–20.2)	14.9 (11.5–20.3)	14.9 (11.1–19.7)
**Business-as-usual (SC2) scenario**									
New child infections due to vertical transmission	4150 (2550–6500)	3100 (1900–4850)	2550 (1600–4050)	2250 (1400–3600)	2050 (1250–3250)	1850 (1150–2950)	1700 (1050–2700)	1550 (950–2450)	1450 (900–2250)
Vertical transmission rate (%)	19.9 (12–30.7)	15.2 (10.4–21.7)	12.9 (10.1–16.5)	11.6 (8.8–14.7)	10.8 (8.4–13.8)	10.1 (7.9–13.3)	9.7 (7.6–13.1)	9.3 (7.2–12.7)	9.0 (6.7–11.9)
**On-track (SC3) scenario**									
New child infections due to vertical transmission	4150 (2550–6500)	3200 (1950–5000)	2700 (1650–4250)	2300 (1400–3600)	1850 (1150–2900)	1450 (900–2300)	1400 (850–2200)	1300 (800–2050)	1250 (750–1950)
Vertical transmission rate (%)	19.9 (12–30.7)	15.7 (10.7–22.4)	13.5 (10.6–17.2)	11.6 (8.8–14.7)	9.7 (7.6–12.4)	7.9 (6.2–10.4)	7.9 (6.1–10.6)	7.9 (6–10.7)	7.7 (5.8–10.3)
**Fast-track (SC4) scenario**									
New child infections due to vertical transmission	4150 (2550–6500)	2950 (1800–4650)	2250 (1400–3550)	1650 (1000–2600)	1550 (950–2450)	1450 (900–2300)	1400 (850–2150)	1300 (800–2050)	1250 (750–1950)
Vertical transmission rate (%)	19.9 (12–30.7)	14.5 (9.9–20.8)	11.4 (8.9–14.5)	8.5 (6.4–10.7)	8.2 (6.4–10.5)	7.9 (6.2–10.4)	7.9 (6.1–10.6)	7.8 (6–10.7)	7.7 (5.8–10.3)

In the status quo scenario (SC1), in which ART treatment and retention rates are kept at the same level as that of the 2022 level, the number of children getting infected with HIV through the vertical route decreased from 3,500 (2,150–5,500) in 2023 to 2,350 (1,450–3,750) in 2030 with vertical transmission rate declining from 17.20% (11.80%-24.60%) to 14.90% (11.10%-19.70%) (Fig 2). Overall, around 23,200 (14,150–36,600) new infections due to vertical transmission will happen if coverage were maintained at the 2022 level. This is around 57.60% lower than the no-intervention scenario (Table 3 and Figs 2–4).

In the business-as-usual scenario (SC2), where treatment and retention gradually increased over the years based on the observed trend in treatment coverage during 2020 and 2022, annual new infections because of vertical transmission decreased from 3,100 (1,900–4,850) in 2023 to 1,450 (900–2,250) in 2030. The vertical transmission rate in the same period decreased from 15.20% (10.40%-21.70%) to 9.00% (6.70%-11.90%). Overall, around 16,500 (10,200–26,100) new infections due to vertical transmission would happen in this business-as-usual scenario, 69.80% lower than the no-intervention scenario and 28.80% lower than the status quo scenario (Table 3 and Figs 2–4).

In the on-track scenario (SC3), where coverage and retention rates were linearly modified to attain 95% by 2027, annual vertical infections decreased from 3,200 (1,950–5,000) in 2023 to 1,250 (750–1,950) in 2030 with vertical transmission rate declining from 15.70% (10.70%-22.40%) to 7.70% (5.80%-10.30%). Cumulatively, around 15,450 (9,450–24,250) new vertical transmissions will happen over 8 years in the ‘on-track’ scenario, lower by 71.80%, 33.60% and 6.70% vis-à-vis no intervention, status quo, and business as usual scenario respectively (Table 3 and Figs 2–4).

In the fast-track scenario (SC4), where coverage and retention rates were linearly modified to attain 95% by 2025, annual vertical infections decreased from 2,950 (1,800–4,650) in 2023 to 1,250 (750–1,950) in 2030 with vertical transmission rate declining from 14.50% (9.90%-20.80%) to 7.70% (5.80%-10.30%). Cumulatively, around 13,800 (8,450–21,700) new vertical transmissions will happen over 8 years in the ‘fast-track’ scenario, lower by 74.70%, 40.40%, 16.30% and 10.30% vis-à-vis no intervention, status quo, business as usual and on-track scenario respectively (Table 3 and Figs 2–4).

## Discussion

The goal of eliminating vertical transmission of HIV is crucial to India’s pledge to end AIDS as a public health threat by 2030. The analysis provided herein demonstrates a marked decrease in vertical transmission, a direct result of the initiatives undertaken through the National AIDS and STD Control Programme of the Government of India. Maintaining the current level of programme coverage is projected to avert approximately 31,600 new infections in children from 2023 to 2030, in contrast to an estimated 54,800 child infections that would have occurred in the absence of any intervention. By maintaining the current ART treatment and retention level, the vertical transmission rate in 2030 would be as high as around 14.90% (11.10%-19.70%), much higher than the target of <5%. Upscaling treatment coverage would be key to taking the country closer to the elimination goals.

Our study suggests that if the programme continues to increase the coverage in line with the recent trends, the vertical transmission rate would be as low as 9.00% (6.70%-11.90%) in 2030 averting around 38,300 new child infections. If every State/UT attains the treatment coverage and retention of 95% by 2027 and maintains the level afterwards, the vertical transmission rate would be at 7.70% (5.80%-10.30%) in 2030 averting around 39,300 child infections. If 95% treatment and retention can be achieved by 2025 and maintained subsequently, the vertical transmission rate would be as low as 7.70% (5.80%-10.30%) in 2030 while averting around 41,000 child infections.

However, even in the most ambitious fast-track scenario, the vertical transmission rate in 2030 would be higher than the target of <5% establishing that attainment of the elimination goal would not be possible through ART alone as transmission through breastfeeding also plays a critical role. Around 40% of the total vertical transmissions in India are estimated to occur in the post-natal period [13,18]. Prolonged breastfeeding beyond 12 months, even when the mother is on ART, is associated with an increased risk of vertical transmission. Given the context, the role of breastfeeding practices in vertical transmission has been heavily debated and suggestions have been made to limit the duration of breastfeeding with ART to 12 months. In selected settings, acceptable, feasible, affordable, sustainable, and safe substitutes for breastfeeding exist, provided that mothers receive proper counselling [23–26]. However, in countries such as India, the total eschewal of breastfeeding represents a complex intervention and is not recommended given its correlation with increased rates of non-HIV morbidity and mortality.

Additional gains in reductions of vertical transmission may be made by augmenting focus on prong 1 of primary prevention and prong 2 of meeting the unmet need for family planning among women living with HIV. The risk of vertical transmission for incident infections during pregnancy or breastfeeding is quite high as extraordinarily high maternal viral load during incident infections would result in more transmission. Around 18% of the total vertical transmissions in India are estimated to be among incident maternal infections, either during pregnancy or breastfeeding [13,18]. While the overall incidence rate in India is already very low, it maintains a declining trajectory and any further progress on primary prevention would further improve the progress on elimination of vertical transmission. Reducing the unmet need for family planning has a role in attainment of the elimination of the vertical transmission of HIV [7,8,12]. Understanding the magnitude and reasons for the unmet need and implementing specific activities to reduce unintended pregnancies will further help NACP in attaining EVTH.

The study indicates a huge gain made by the National AIDS and STD Control Programme in India. Studies conducted in various regions have demonstrated that preventing vertical transmissions is highly cost-effective, yielding significant savings in both direct and indirect costs for families and society at large [27–30]. The return on investment has been noted to be as high as 88.4, indicating that every US$ 1 invested yielded approximately US$ 88.4 in benefits [31]. An investment of US$ 49 million in a PMTCT (Prevention of Mother-To-Child Transmission) program in another region successfully averted an estimated 2,725 new pediatric HIV infections. This intervention resulted in an estimated savings of US$ 0.5 billion in treatment expenditures for the healthcare system and nearly US$ 3.9 billion in productivity gains.

### Limitations

Our analysis provides an opportunity to understand the potential impact of the scaling up of the ART treatment coverage among HIV-positive women on the estimated number of vertical transmissions during 2023–2030 from the 2022 round of HIV burden estimations in India. However, there are limitations which need to be considered to put the results in context and have been described previously, in general as well as in the context of estimation of vertical transmission of HIV in India [18,32].

In brief, limitations are inherent to the Spectrum model but also arise from input data. Many assumptions on the fertility rates and transmission probabilities are based on the global cohort studies with limited to no study sites in India. There is limited evidence on the validation of assumptions and modelled vertical transmission infections and rates. Also, the model considers vertical transmission as the only mode of transmission in the pediatric population and does not take into account the potential horizontal transmission. Scarce India-specific data on ART adherence and retention among HIV-positive mothers as well as breastfeeding patterns also contributes to limitations.

## Conclusion

Despite the limitations, to the best of our knowledge, the analysis presented in this paper is the first of its kind to quantify the potential impact of the scaling-up of the treatment coverage among vertical transmission in India. The analysis establishes that India is on track to attain the elimination of vertical transmission of HIV as a core component of the 2030 goal of ending AIDS as a public health threat by 2030. Fast-tracking scale-up of treatment services would avert almost 41000 child infections vis-à-vis no intervention scenario with a vertical transmission rate of around 7.70% (5.80%-10.30%) in 2030. Coupled with ongoing programmatic efforts on primary prevention and reduction of unmet need for family planning, evidence indicates that the country is set to attain the elimination of vertical transmission on or before 2030. As the attainment of vertical transmission of HIV will take an enormous amount of effort and resources from the government, the quantification of the potential impact would provide insights not only into the progress on the committed targets but also into the return of investment from a financial perspective. Sustained focus on and evolution of availability of EVTH-related strategic information under NACP as an overarching pillar through the complementing systems of programme monitoring, surveillance & epidemiology and research is required to augment the country’s progress on elimination of vertical transmission.

## Supporting information

S1 Table ART treatment coverage and retention rate during the projection period of 2023 to 2036, status quo scenario (SC1). (PDF)

S2 Table ART treatment coverage and retention rate during the projection period of 2023 to 2036, business as usual scenario (SC2). (PDF)

S3 Table ART treatment coverage and retention rate during the projection period of 2023 to 2036, on-track scenario (SC3). (PDF)

S4 Table ART treatment coverage and retention rate during the projection period of 2023 to 2036, fast-track scenario (SC4). (PDF)

## References

[1] United Nations. Sustainable Development Goals. Goal 3: Ensure healthy lives and promote well-being for all at all ages. Available from: http://www.un.org/sustainabledevelopment/health/.

[2] United Nations General Assembly. Political Declaration On HIV And AIDS: On The Fast-Track To Accelerate The Fight Against HIV And To End The AIDS Epidemic By 2030. United Nations; 2016. Available from: https://www.unaids.org/en/resources/documents/2016/2016-political-declaration-HIV-AIDS.

[3] World Health Organization. Global guidance on criteria and processes for validation: Elimination of Mother-to-child Transmission of HIV, Syphilis and Hepatitis B Virus. World Health Organization; 2021.

[4] Goga A, Singh Y, Jackson D, Pillay Y, Bhardwaj S, Chirinda W, et al. Is elimination of vertical transmission of HIV in high prevalence settings achievable? bmj. 2019 Mar 26;364. doi: 10.1136/bmj.l687 30957782 PMC6434516

[5] World Health Organization. Countries which have received WHO validation. WHO, Geneva 2022. Available from: https://www.who.int/initiatives/triple-elimination-initiative-of-mother-to-child-transmission-of-hiv-syphilis-and-hepatitis-b/validation.

[6] Elgalib A, Lau R, Al-Habsi Z, Shah S, Al-Rawahi B, Memish ZA, et al. Elimination of mother-to-child transmission of HIV, syphilis and viral hepatitis B: A call for renewed global focus. International Journal of Infectious Diseases. 2023 Feb 1;127:33–5. doi: 10.1016/j.ijid.2022.11.031 36574535

[7] Chi BH, Mbori‐Ngacha D, Essajee S, Mofenson LM, Tsiouris F, Mahy M, Luo C. Accelerating progress towards the elimination of mother‐to‐child transmission of HIV: a narrative review. Journal of the International AIDS Society. 2020 Aug;23(8):e25571. doi: 10.1002/jia2.25571 32820609 PMC7440973

[8] UNICEF, UNAIDS W. Key considerations for programming and prioritization. going the ‘LAST MILE’to EMTCT: A road map for ending the HIV epidemic in children 2020.

[9] Kumar P, Das C. Elimination of Vertical Transmission of HIV and Syphilis as a Public Health Threat in India: Strategic Information-Driven Pathway. Indian Journal of Community Medicine: Official Publication of Indian Association of Preventive & Social Medicine. 2022 Oct;47(4):467. doi: 10.4103/ijcm.ijcm_905_22 36742981 PMC9891059

[10] National AIDS Control Organization. Strategy Document: National AIDS and STD Control Programme Phase-V (2021–26). New Delhi: NACO, Ministry of Health and Family Welfare, Government of India; 2022.

[11] National AIDS Control Organisation Updated Guidelines for Prevention of Parent to Child Transmission (PPTCT) of HIV using Multi-Drug Anti-retroviral Regimen in India. New Delhi: National AIDS Control Organization, Ministry of Health &Family Welfare, Government of India 2013.

[12] Mahy M, Stover J, Kiragu K, Hayashi C, Akwara P, Luo C, et al. What will it take to achieve virtual elimination of mother-to-child transmission of HIV? An assessment of current progress and future needs. Sexually transmitted infections. 2010 Dec 1;86(Suppl 2):ii48–55. doi: 10.1136/sti.2010.045989 21106515 PMC3173823

[13] National AIDS Control Organization. Sankalak: Status of National AIDS Response. Fifth edition. New Delhi: NACO, Ministry of Health and Family Welfare, Government of India; 2023.

[14] National AIDS Control Organization & ICMR-National Institute of Medical Statistics. India HIV Estimates 2022: Report. New Delhi: NACO, Ministry of Health and Family Welfare, Government of India. 2023.

[15] Rollins N, Mahy M, Becquet R, Kuhn L, Creek T, Mofenson L. Estimates of peripartum and postnatal mother-to-child transmission probabilities of HIV for use in Spectrum and other population-based models. Sexually transmitted infections. 2012 Dec 1; 88(Suppl 2):i44–51. doi: 10.1136/sextrans-2012-050709 23172345 PMC3512432

[16] Lewis JJ, Ronsmans C, Ezeh A, Gregson S. The population impact of HIV on fertility in sub-Saharan Africa. Aids. 2004 Jun 1; 18:S35–43. doi: 10.1097/00002030-200406002-00005 . 15319742

[17] Chen WJ, Walker N. Fertility of HIV-infected women: insights from Demographic and Health Surveys. Sexually transmitted infections. 2010 Dec 1; 86(Suppl 2):ii22–7. doi: 10.1136/sti.2010.043620 . 21106511 PMC3173817

[18] Kumar P, Das C, Das U, Kumar A, Priyam N, Ranjan V, et al. Augmenting progress on the elimination of vertical transmissions of HIV in India: Insights from Spectrum-based HIV burden estimations. PLOS Global Public Health. 2023 Aug 9;3(8):e0002270. doi: 10.1371/journal.pgph.0002270 37556441 PMC10411776

[19] National AIDS Control Organization. Integrated and Enhanced Surveillance and Epidemiology of HIV, STI and related Co-morbidities Under the National AIDS and STD Control Programme: Strategic Framework. NACO, Ministry of Health and Family Welfare, Government of India; 2022.

[20] Pandey A, Reddy DC, Ghys PD, Thomas M, Sahu D, Bhattacharya M, et al. Improved estimates of India’s HIV burden in 2006. Indian Journal of Medical Research. 2009 Jan 1; 129(1):50–8. . 19287057

[21] Sahu D, Kumar P, Chandra N, Rajan S, Shukla DK, Venkatesh S, et al. Findings from the 2017 HIV estimation round & trend analysis of key indicators 2010–2017: Evidence for prioritising HIV/AIDS programme in India. Indian Journal of Medical Research. 2020 Jun 1;151(6):562. doi: 10.4103/ijmr.IJMR_1619_19 . 32719229 PMC7602920

[22] Kumar P, Das C, Kumar A, Sahu D, Rai SK, Godbole S, et al. Diversity in HIV epidemic transitions in India: An application of HIV epidemiological metrices and benchmarks. Plos one. 2022 Jul 18;17(7):e0270886. doi: 10.1371/journal.pone.0270886 35849570 PMC9292090

[23] Stover J, Fidzani B, Molomo BC, Moeti T, Musuka G. Estimated HIV trends and program effects in Botswana. PLoS One. 2008 Nov 14;3(11):e3729. doi: 10.1371/journal.pone.0003729 19008957 PMC2579326

[24] Creek TL, Kim A, Lu L, Bowen A, Masunge J, Arvelo W, et al. Hospitalization and mortality among primarily nonbreastfed children during a large outbreak of diarrhea and malnutrition in Botswana, 2006. JAIDS Journal of Acquired Immune Deficiency Syndromes. 2010 Jan 1;53(1):14–9. doi: 10.1097/QAI.0b013e3181bdf676 19801943

[25] Horvath T, Madi BC, Iuppa IM, Kennedy GE, Rutherford GW, Read JS. Interventions for preventing late postnatal mother‐to‐child transmission of HIV. Cochrane database of systematic reviews. 2009(1). doi: 10.1002/14651858.CD006734.pub2 19160297 PMC7389566

[26] Young SL, Mbuya MN, Chantry CJ, Geubbels EP, Israel-Ballard K, Cohan D, et al. Current knowledge and future research on infant feeding in the context of HIV: basic, clinical, behavioral, and programmatic perspectives. Advances in Nutrition. 2011 May 1;2(3):225–43. doi: 10.3945/an.110.000224 22332055 PMC3090166

[27] Söderlund N, Zwi K, Kinghorn A, Gray G. Prevention of vertical transmission of HIV: analysis of cost effectiveness of options available in South Africa. Bmj. 1999 Jun 19;318(7199):1650–6. doi: 10.1136/bmj.318.7199.1650 10373166 PMC28142

[28] Binagwaho A, Pegurri E, Drobac PC, Mugwaneza P, Stulac SN, Wagner CM, et al. Prevention of mother-to-child transmission of HIV: cost-effectiveness of antiretroviral regimens and feeding options in Rwanda. PloS one. 2013 Feb 20;8(2):e54180. doi: 10.1371/journal.pone.0054180 23437040 PMC3577801

[29] Patrikar S, Bhardwaj M, Dudeja P, Kunte R. Cost-effectiveness of the prevention of parent-to-child transmission guidelines of HIV in India. Medical Journal Armed Forces India. 2022 Nov 18. doi: 10.1016/j.mjafi.2022.09.002 38800004 PMC11116979

[30] Qu SL, Wang AL, Yin HM, Deng JQ, Wang XY, Yang YH, et al. Cost-effectiveness analysis of the prevention of mother-to-child transmission of HIV. Infectious Diseases of Poverty. 2022 Jun 15;11(1):68. doi: 10.1186/s40249-022-00983-z 35706049 PMC9202156

[31] Wang X, Guo G, Zheng J, Lu L. Programmes for the prevention of mother-to-child HIV infection transmission have made progress in Yunnan Province, China, from 2006 to 2015: a cost effective and cost-benefit evaluation. BMC Infectious Diseases. 2019 Dec;19:1–1. doi: 10.1186/s12879-019-3708-x 30654744 PMC6337853

[32] Penazzato M, Bendaud V, Nelson L, Stover J, Mahy M. Estimating future trends in paediatric HIV. AIDS. 2014 Nov 1;28:S445–51. doi: 10.1097/QAD.0000000000000481 25409099 PMC4247271

