# Supplementary material for: Projected impact of fast-tracking of anti-retroviral treatment coverage on vertical transmission of HIV in India
Source: PLOS Glob Public Health. 2024 Sep 18;4(9):e0003702. doi: 10.1371/journal.pgph.0003702 (PMC11410226; doi:10.1371/journal.pgph.0003702)
Supplement: S4 Table — (PDF) [file pgph.0003702.s004.pdf]

Supplementary Table S4. ART treatment coverage and retention rate during the projection period of 2023 to 2036, fast-track scenario (SC4)

Subnational region	2022	2023	2024	2025	2026	2027	2028	2029	2030
Andhra Pradesh (AP)	86	89	92	95	95	95	95	95	95
Arunachal Pradesh (AR)	83	87	91	95	95	95	95	95	95
Assam (AS)	54	68	81	95	95	95	95	95	95
Bihar (BH)	27	50	72	95	95	95	95	95	95
Chhattishgarh (CG)	74	81	88	95	95	95	95	95	95
DElhi (DL)	78	84	89	95	95	95	95	95	95
Goa (GO)	89	91	93	95	95	95	95	95	95
Gujarat (GJ)	86	89	92	95	95	95	95	95	95
Himachal Pradesh (HP)	66	76	85	95	95	95	95	95	95
Haryana (HR)	88	90	93	95	95	95	95	95	95
Jharkhand (JH)	71	79	87	95	95	95	95	95	95
Jammu & Kashmir (JK)	44	61	78	95	95	95	95	95	95
Karnataka (KA)	92	93	94	95	95	95	95	95	95
Kerala (KE)	91	92	94	95	95	95	95	95	95
Meghalaya (MG)	88	90	93	95	95	95	95	95	95
Maharashtra (MH)	83	87	91	95	95	95	95	95	95
Manipur (MN)	51	65	80	95	95	95	95	95	95
Madhya Pradesh (MP)	95	95	95	95	95	95	95	95	95
Mizoram (MZ)	85	88	92	95	95	95	95	95	95
Nagaland (NG)	67	76	86	95	95	95	95	95	95
Odisha (OD)	61	72	84	95	95	95	95	95	95
Punjab (PJ)	95	95	95	95	95	95	95	95	95
Rajasthan (RJ)	91	92	94	95	96	96	96	96	96
Sikkim (SK)	87	90	92	95	95	95	95	95	95
Tamil Nadu (TN)	89	91	93	95	95	95	95	95	95
Tripura (TR)	72	80	87	95	96	96	96	96	96
Uttarakhand (UK)	37	56	76	95	95	95	95	95	95
Uttar Pradesh (UP)	83	87	91	95	95	95	95	95	95
West Bengal (WB)	89	91	93	95	95	95	95	95	95
Andaman & Nicobar (AN)	56	69	82	95	95	95	95	95	95
Chandigarh (CH)	95	95	95	95	95	95	95	95	95
Dadra & Nagar	61	72	84	95	95	95	95	95	95
Pondicherry (PO)	35	55	75	95	95	95	95	95	95
Telegana (TL)	90	92	93	95	96	96	96	96	96
